# Endoscopic hand suturing with muscle anchoring for complete defect closure after endoscopic submucosal dissection

**DOI:** 10.1055/a-2860-0486

**Published:** 2026-05-08

**Authors:** Oscar Cahyadi, Mousa Ayoub, Sarah Schellen, Felix Gartung, Esma Zeynep Kızılaslan, Alanna Ebigbo

**Affiliations:** 1 Department of Gastroenterology 91789 St. Josef-University Hospital BochumBochumGermany; 2 74889 Ruhr University of BochumBochumGermany

Endoscopic submucosal dissection (ESD) is the primary therapeutic modality for the treatment of early gastric cancer 1 . In patients under oral anticoagulation and with increasing age and comorbidities, defect closure after ESD can reduce secondary complications such as bleeding and delayed perforation 2 3 . Endoscopic hand suturing (EHS) for defect closure has emerged as a novel closure modality and 4 utilizes an endoscopic needle holder (FG-260, Olympus, Tokyo, Japan) and a resorbable barbed suture (V-Loc 180, Covidien, Mansfield, USA). In the stomach, defect closure can be challenging. EHS with mucosa-to-mucosa sutures may not lead to complete defect closure and may create a dead space below the mucosal suture. We propose a technique of EHS with additional muscle anchoring stitches to facilitate complete closure ( Video 1 ). With this technique, fixating the barbed suture to the muscle facilitates defect closure, especially in large defects.

This video shows the muscle anchoring technique during the endoscopic hand suturing process to facilitate the complete closure of a wide defect after endoscopic submucosal dissection in the subcardiac region.Video 1

**Fig. 1:**
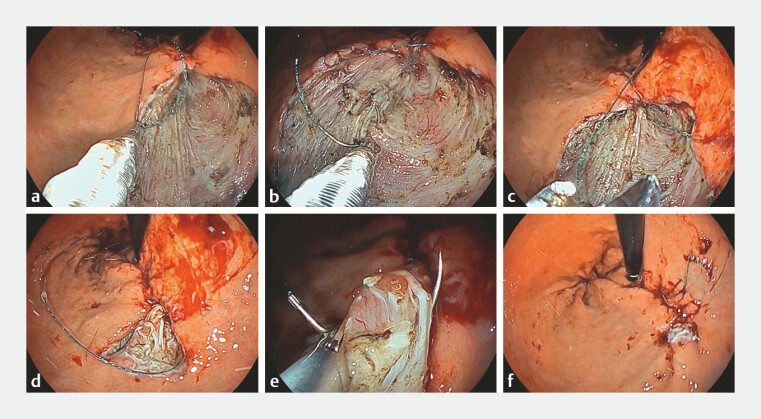
Example of EHS with muscle anchoring to close a large defect: **a** after the first mucosal-stich placement continuation of EHS in retroflection, **b** the placement of a muscle anchor to bridge the large gap, **c** the gap was not completely approximated, since the suture was not yet tightened – this highlights the importance of tightening the suture frequently, **d** multiple muscle anchoring followed by suture tightening led to a tight closure, **e** final muscle anchoring before the last stitch and **f** the final image of the defect with a complete closure. EHS, endoscopic hand suturing.

The patient was an 84-year-old man with chronic kidney and heart failure and under anti-platelet therapy. He was referred for endoscopic resection of a 35 mm subcardiac gastric carcinoma. ESD was successful, and closure with EHS was performed to avoid secondary complications. The first stitch was placed in the antegrade scope position but the remaining EHS was completed in retroflection ( Fig. 1 **a** ). During EHS, complete closure was not possible by mucosa-to-mucosa approximation alone despite tightening the suture multiple times. Thus muscle-anchoring of the suture was pursued by passing the suture directly through the muscle layer ( Fig. 1 **b–e** ). Complete closure was achieved after performing multiple running sutures with muscle anchoring ( Fig. 1 **f** ). Notably, no complications occurred during the procedure. Oral intake could be resumed after the ESD and further post-ESD course was uneventful. He was discharged on the second post-ESD day. We believe that this technique is useful to improve the closure of large defects, especially in the stomach.

Endoscopy_UCTN_Code_TTT_1AO_2AG
